# Optimized Clarification Technology of Bayberry Juice by Chitosan/Sodium Alginate and Changes in Quality Characteristics during Clarification

**DOI:** 10.3390/foods11050671

**Published:** 2022-02-24

**Authors:** Andi Wu, Jimin Lv, Changxin Ju, Yiwen Wang, Yanyun Zhu, Jianchu Chen

**Affiliations:** College of Biosystems Engineering and Food Science, Zhejiang University, Hangzhou 310058, China; 21913081@zju.edu.cn (A.W.); lvjimin@zju.edu.cn (J.L.); 21913065@zju.edu.cn (C.J.); 21913039@zju.edu.cn (Y.W.); 11813043@zju.edu.cn (Y.Z.)

**Keywords:** bayberry juice, clarification, chitosan/sodium alginate, composite clarifiers, aroma

## Abstract

In this study, a novel method to clarify bayberry juice with composite clarifiers, chitosan and sodium alginate, has been designed. The optimal conditions were as follows: using chitosan 0.05 g/L first and then sodium alginate 0.05 g/L as composite clarifiers, standing for 2 h at 25 °C. The transmittance increased from 0.08 to 91.2% after treating by composite clarifiers, which was significantly higher than using chitosan (44.29%) and sodium alginate (38.46%) alone. It was also found that sedimentation time of juice treated by composite clarifiers was about 60% shorter than using single clarifiers. Meanwhile, the reduction of anthocyanin in juice was 9.16% for composite clarifiers treatment, being less than that for the single sodium alginate and previous related researches. In addition, the color and aroma of bayberry juice treated by composite clarifiers were improved. Juice treated by composite clarifiers had the highest L* value with 52.48 and looked more attractive. The present research revealed that content of beta-damascenone and dihydro-5-pentyl-2(3H)-furanone increased after treatment with composite clarifiers which contributed more to the pleasant aroma. Overall, the developed method improved the clarification effect and sensory quality, and reduced the sedimentation time, which may be promising in the production of clear bayberry juice.

## 1. Introduction

Chinese bayberry (Myrica rubra Sieb. et Zucc.) is a very popular fruit in the south of China with its pleasant color, wonderful aroma and unique sweet–sour taste [1]. Since rich in phenolic compounds, Chinese bayberry is considered as a highly nutritious fruit [2]. However, without the protection of exocarp, it has a short maturation period and poor storage stability. Processing into fruit beverage is an effective way to avoid spoilage and waste of bayberry, which can extend the shelf-life of bayberry. 

In the process of pressing, however, pectins, tannins and polysaccharides are dispersed in juice, and these substances interact with each other, resulting in the performance of turbidity and sedimentation in the juice [3]. In order not to affect consumer acceptance of juice, most fruit beverages require clarification during processing to avoid unnecessary turbidity, haze, and sediments in the final products [4]. Polysaccharide-based clarifying agents, such as β-cyclodextrin [3], chitosan (CTS) [5], sodium alginate (SA) [6] could be used to produce clear fruit juice. However, there are some disadvantages, for instance long reaction/sedimentation time or poor clarification effect without centrifugation when using a kind of clarifier alone. For achieving better results, previous studies have shown the application of composite clarifiers in juice processing. Calcium alginate/CTS/polydopamine composite beads retained the clarifying ability of CTS while improving the stability during the clarification process of apple juice [7]. Tannin acid/CTS showed higher retention rate of vitamin C and transmittance than that for the CTS treatment alone on clarification of kiwifruit juice [8]. 

The properties and characteristics of different clarifiers have a marked impact on the clarification effect [6]. CTS is a natural cationic polymer with primary amine groups and SA is an anionic polymer with polar groups such as -COO^−^ [9]. Electronic interactions play a fundamental role in the formation of CTS/SA precipitation, since CTS and SA are polyelectrolytes with oppositely charges. CTS/SA could be an excellent amphoteric absorbent because both positively and negatively charged compounds might be absorbed by CTS/SA, then was removed from the solution in the process of CTS/SA precipitation [10,11]. Studies have shown that oppositely charged dyes Rhodamine B and Acid Blue-113 could be removed in wastewater by composite foams using CTS/SA as materials simultaneously [12]. Generally, the CTS/SA had many advantages, such as excellent adsorption capacity, high efficiency, low pH sensitivity and environmental friendliness, which were used in dye wastewater [13], contaminants [14] and microalgae treatment [15]. CTS/SA may be effective in clarifying bayberry juice, because there are negative constituents in bayberry juice, such as protein and pectin, as well as positive compounds, such as anthocyanin. However, the application of CTS/SA composite clarifiers in fruit juice clarification has not yet been studied.

In this work, bayberry juice was used as a representative fruit juice to investigate the clarification performance of CTS/SA, so as to fill the gap in the application of CTS/SA in fruit juice treatment. The objectives of this study were to find the optimal parameters for clarification using CTS/SA and to evaluate the effect of different clarification methods on protein, pectin, anthocyanin, color parameters, aroma, etc. Therefore, the effects of adding sequence, standing time, temperature, dosage, volume ratio and pH on the clarification of bayberry juice were investigated and then optimized. Gas chromatography–mass spectrometry (GC-MS) was used to detect the volatile flavor components, while aroma variation was researched by multiple analytical methods. Moreover, the study attempted to elucidate the clarification mechanisms of CTS/SA by analyzing correlation between transmittance and the content of main compositions.

## 2. Materials and Methods

### 2.1. Chemicals

CTS was purchased from Macklin (Shanghai, China); SA was purchased from Henan Wan Bang Industrial Co., Ltd., Zhengzhou, China; phenol, sulfuric acid, glucose, sodium hydroxide, carbazole, sodium chloride and ethanol were purchased from Shanghai Chemical Reagent Co. (Shanghai, China); cyclohexanone and formic acid were purchased from Aladdin (Shanghai, China); methanol was purchased from TEDIA (Anhui, China); rutin and anthocyanins were purchased from Solarbio (Beijing, China).

### 2.2. Processing Methods

#### 2.2.1. Chinese Bayberry Juice

Chinese bayberry cultivars of Biqi (M.rubra Sieb. et Zucc.) were collected from Cixi, China, in June 2020 and stored at −20 °C. Thawed frozen bayberry fruit at room temperature and then squeezed to juice. A 200-mesh nylon bag was used to separate the pomace from juice.

The processing and clarification of bayberry juice were shown in Figure S1. Raw bayberry juice stirred at a low speed at 25 °C for 2 h was used as control sample (unclarified). The clarification method of bayberry juice held at 25 °C for 2 h without stirring was named natural sedimentation method (NS).

#### 2.2.2. Clarification by Individual Clarifiers

The solvents of 1% (*w/v*) CTS solution and 1% (*w/v*) SA were acetic acid aqueous solution and distilled water, respectively. In order to discuss the optimum doses of individual clarifiers, the concentration of (0.1, 0.2, 0.3, 0.4, 0.5 g/L) of CTS and (0.0125, 0.025, 0.05, 0.1, 0.2 g/L) of SA were determined. And the mixture was stirred by electric mixer (TECNICA, YIDA-XINYI-612, China) at 200 r/min for 3 min. Better repeatability could be obtained under the fixed stirring speed and time. After incubation at 25 °C for 2 h, the supernatant was collected for analysis. The transmittance at 750 nm determined with a spectrophotometer (Shimadzu, UV-2550, Japan) was the index to determine the clarification effect (the same below).

#### 2.2.3. Clarification by Composite Clarifiers

Single-factor experiment of clarification conditions:

In order to improve transmittance and clarified efficiency, the treatment by two clarifiers of CTS and SA were carried out. The parameters including addition sequence of CTS and SA (A: CTS was added into juice first followed by the addition of SA (i.e., CTS/SA), B: oppositely, CTS was added into juice after SA. For sequence A and B, the system was stirred for 2 min after adding the former clarifier and stirred for 1 min after adding the latter. C: CTS and SA were added at the same time and stirred for 3 min. The stirring speed of the above was all 200 r/min), standing time (1, 2, 3, 4, 5 h), incubation temperature (4, 15, 25, 35, 45 °C), total concentration (0.05, 0.1, 0.2, 0.3, 0.4 g/L), volume ratio of CTS to SA (1:4, 1:3, 1:1, 3:1, 4:1), pH of juice (3.0, 3.2, 3.4, 3.6, 3.8) were conducted to screen the factors and levels for orthogonal test.

Screen for optimal clarification conditions by orthogonal test:

Based on the single-factor experiment results, the volume ratio of CTS to SA (A), the total concentration (B) and the incubation temperature (C) were identified as key factors, the transmittance as the evaluation index. The factors and levels were as seen in Table S1.

Due to the effect of CTS/SA was the result of the joint clarification of NS and CTS/SA. To exclude the influence of NS clarification, the control group was set. Raw bayberry juice was held at 25 °C for 2 h, then the supernatant was taken out and treated with CTS/SA under the optimum process conditions. We term the clarification method ‘NS/CTS/SA’ for simplicity.

### 2.3. Sedimentation Time and Final Turbidity Volume

During sedimentation of flocs, a clear-turbidity level exists in sample. Above the level is the supernatant and below is the sedimented flocs. Juice (40 mL) without standing process was taken in 50 mL graduated tubes and the time was recorded. The time needed for clear-turbidity level from 40 mL to reach 20 mL was the sedimentation time, which could reflect the clarified velocity of flocs and clarified efficiency. The shorter the time, the faster the velocity, so as to reduce nutrient loss or spoilage during clarification. The final turbidity volume (i.e., the total volume of flocs) which was obtained directly in the graduated tube at the end of the clarification process, reflected the yield of the clarified juice to a certain extent. The larger the final turbidity volume of flocs, the smaller supernatant volume and the lower the yield of clarified juice.

### 2.4. Determination of Physicochemical Parameters

#### 2.4.1. Protein Determination (Bradford Assay)

The content of protein was measured according to the method described in the Bradford Protein Assay kit (Beyotime, Haimen, China). The absorbance was measured at 765 nm with BSA as the standard compound.

#### 2.4.2. Total Soluble Solids and Total Carbohydrate Content

Determination of total soluble solids was performed by a pocket Brix-Acidity meter (PAL-BXIACID F5, Atago Co. Ltd., Tokyo, Japan) as previously described [6]. The carbohydrate content was analyzed, according to phenol-H_2_SO_4_ colorimetric method described by Zhu et al. [1] and Son et al. [16], with some modifications. The juice sample (2 μL) was diluted in 1998 μL water, then mixed with 1 mL phenol solution (6% *v/v*) and 5 mL sulfuric acid. The mixture was incubated at 100 °C for 15 min. The absorbance values were measured at 490 nm after the mixture cooling to room temperature.

#### 2.4.3. Tannin Content, Total Phenolic Content, Total Flavonoids Content and Total Anthocyanins Content

The Folin–Ciocalteu assay was used to determine total phenolics [1] and the results were reported as milligrams gallic acid equivalents (GAE) per liter of juice. The total flavonoids content was measured by UV/Vis spectrophotometric method [1]. The measurement of the absorbance was at 510 nm with rutin as the standard. The tannin content was measured according to Zhong et al. [17], with some modifications. The sample (50 μL) was mixed with 2 mL Folin–Denis reagent and 10 mL saturated NaCO_3_ in 50 mL volumetric flask, and diluted with water to volume. After 30 min incubation at room temperature, the absorbance was determined at 720 nm with tannin as standard. Total anthocyanins content was analyzed by applying the pH differential method, according to Carabarin et al. [18]. Results were expressed as mg of cyanidin-3-glucoside equivalents per liter of juice.

#### 2.4.4. Pectin Content

The pectin content of juice was measured using the method of Tastan et al. [5] with minor modification. 15 mL samples and 25 mL ethanol (95% *v/v*) were incubated at 85 °C for 10 min. Then sample was centrifugated (3000 r/min, 15 min) and washed with ethanol (63% *v/v*). After that, the sediment was treated with 5 mL of 1 M NaOH. The sample was filtered and the filtrate was collected for analysis after standing for 15 min. A total of 1 mL filtrate, 0.5 mL carbazole–alcohol solution (0.1%), and 6 mL sulfuric acid were added into a tube, then were incubated at 85 °C for 5 min. The absorbance was measured at 525 nm and D-Galacturonic acid was as the standard. 

#### 2.4.5. Color Analysis

A tristimulus colorimetric (ColorFlex EZ, Hunterlab, Reston, VA, USA) was used to determine the tristimulus CIE values (L*, a*, b*). The total color differences (Δ*E*) of samples were calculated based on the following equations:

$$\Delta E=\sqrt{{\left(L^{*}-L_{0}^{*}\right)}^{2}+{\left(a^{*}-a_{0}^{*}\right)}^{2}+{\left(b^{*}-b_{0}^{*}\right)}^{2}}$$

where the color parameters subscripts with “0″ were color values of unclarified bayberry juice samples.

### 2.5. Determination of Aroma Using GC-MS

Headspace solid-phase microextraction (HS-SPME) was used for volatile extraction of bayberry juice [19]. A pretreated SPME fiber (DVB/CAR/PDMS, Supelco, PA, USA) was equilibrated for 15 min at 50 °C, then was used to extract the sample for 30 min at 50 °C. The fiber was introduced into the injection port of Agilent 7890A gas chromatograph coupled with an Agilent 5975C mass selective detector, and was desorbed at 240 °C for 5 min in spitless mode. A DB-WAX column (30 m × 0.25 mm, 0.25 μm film thickness; Agilent Technologies, Santa Clara, CA, USA) was used to separate volatiles and the pure helium as column carrier gas was at a flow rate of 1.5 mL/min. The source temperature was 280 °C with a mass range of m/z 45–400 amu in the EI mode at 70 eV. The initial oven temperature was held at 60 °C for 5 min, increased to 180 °C at a rate of 5 °C/min, then was ramped up at 10 °C/min to 240 °C and finally held there for 4 min.

Identification of compound was accomplished by matching mass spectral with the standard NIST11 and retention indices (RIs) reported in the AromaChem library of Alpha MOS. A mixture of n-alkanes (C8–C20) was used to calculate the RIs and was matched with those recorded in AromaChem library. Cyclohexanone (1.19 μg/mL) was as internal standard to quantify the identified compounds.

The relative odor activity value (ROAV) is used to evaluate contribution of single compounds to the overall odor [20]. The ROAV_α_ was calculated based on the following equation:

$${\mathrm{ROAV}}_{\alpha }\;\approx 100\times \frac{C{\%}_{\alpha }}{C{\%}_{max}}\times \frac{T_{max}}{T_{\alpha }}\;$$

where *C%_α_* was the relative content of volatile compound α in the sample (%) and *T_α_* was the odor threshold concentration of volatile compound α (μg/kg). *C%_max_* and *T_max_* were the maximum value of *C%_α_*/*T_α_* among all the volatiles in each sample.

The volatiles which ROAV > 1 are considered to be the key odor components of the sample and the volatiles with 0.1 < ROAV < 1 could play an important role in modifying the overall odor.

### 2.6. Statistics Data Analysis

All experiments were performed in triplicate, and the data were expressed as mean ± SD. Statistical analysis and graphics were conducted by Excel for Windows, one-way ANOVA with Duncan’s multiple comparison test at a significance level of 0.05 and Origin 9.5 with principal component analysis (PCA) software.

## 3. Results and Discussion

### 3.1. Effect of Individual Clarification

**Figure 1:**
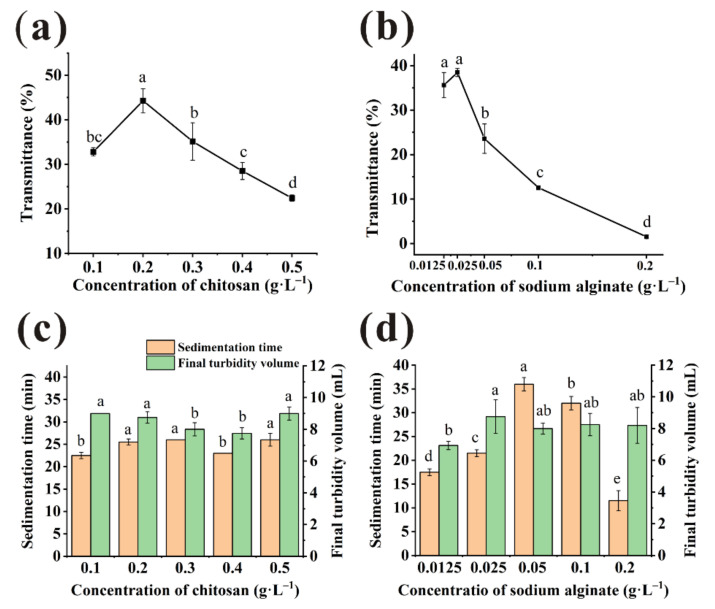
Transmittance of the bayberry juice clarified with chitosan (CTS) (**a**) and sodium alginate (SA) (**b**) under various concentration. Sedimentation time and final turbidity volume of the bayberry juice clarified with CTS (**c**) and SA (**d**) under various concentration. (Different little letters mean a significant difference at the level of 0.05).

As shown in Figure 1a, the highest transmittance of juice treated by CTS was obtained at 0.2 g/L (44.29 ± 2.70%) and higher than unclarified juice samples (0.08 ± 0.00%) significantly. The transmittance of samples rose quickly when the CTS dosage used from 0.1 to 0.2 g/L. However, as the dosage of chitosan increased, the aggregation and precipitation of colloidal particle were limited due to the steric hindrance caused by excessive cationic charge [21,22]. Therefore, the transmittance of bayberry juice decreased when CTS dosage was more than 0.2 g/L. The minimum sedimentation time and flocs volume of CTS were obtained at 0.4 g/L (Figure 1c). The fast velocity would be detrimental to the removal of tiny particles [23], which led to the poor transmittance at 0.4 g/L. Considering that improving the transmittance was the main aim of clarification, 0.2 g/L was selected as the optimal concentration of CTS finally.

Figure 1b showed the transmittance of bayberry juice under varied dosages of SA. The transmittance of juice clarified by 0.0125 g/L and 0.025 g/L SA were 35.61% and 38.46%, respectively. Although two results were statistically similar, it was observed that the small floccules floating on the juice surface after clarification by SA at 0.0125 g/L was much more than that at 0.025 g/L, which affected the clarification effect. When the supernatant was collected to measure transmittance, the floating floccules were collected together sometimes. It was one of the reasons why the transmittance error of juice treated by 0.0125 g/L was higher than 0.025 g/L. Sedimentation time and the final turbidity volume were about 20 min and 9 mL, respectively, when treated by 0.025 g/L SA (Figure 1d). Finally, 0.025 g/L was selected as the optimal concentration of SA.

### 3.2. Effect of Composite Clarifiers

#### 3.2.1. The Adding Sequence of Clarifiers

**Figure 2:**
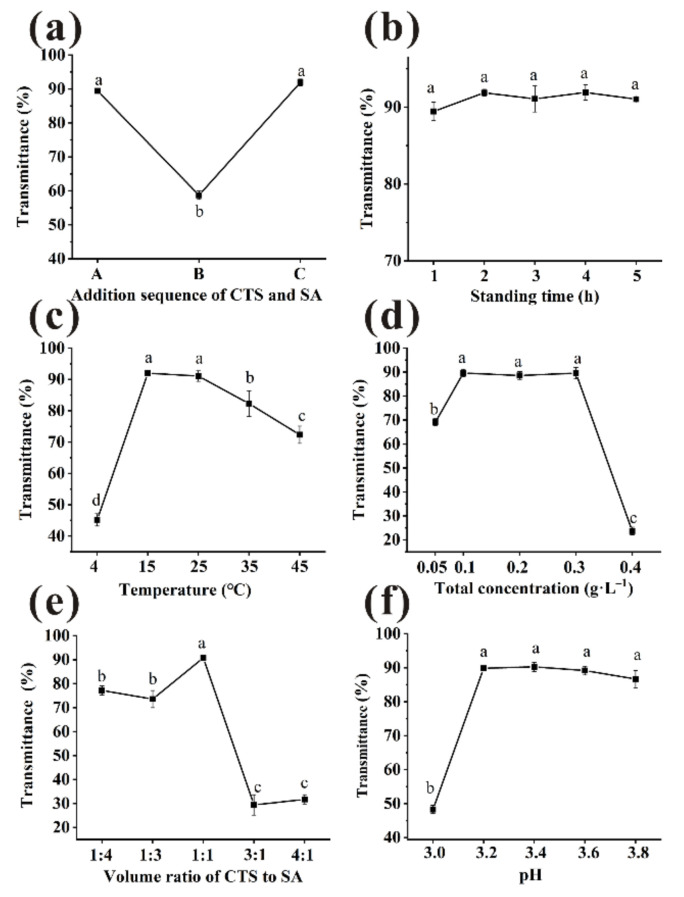
Various factors ((**a**): addition sequence, (**b**): standing time, (**c**): temperature, (**d**): total concentration, (**e**): volume ratios, (**f**): pH) to influence the transmittance of bayberry juice. (Different little letters mean a significant difference at the level of 0.05).

Floc formation is affected by the addition sequence of clarifiers, thus affecting flocculation and clarification [24]. The composite clarifiers with total concentration of 0.2 g/L (CTS:SA = 1:1, *v/v*) in three sequences were used, and incubated at 25 °C for 3 h. Sequence A led to a significant increase in beverage transmittance compared with sequence B, from 58% to 90% (Figure 2a). No statistical differences (*p* > 0.05) were observed between sequence A and C, which were 89.42% and 91.91%, respectively. 

**Figure 3:**
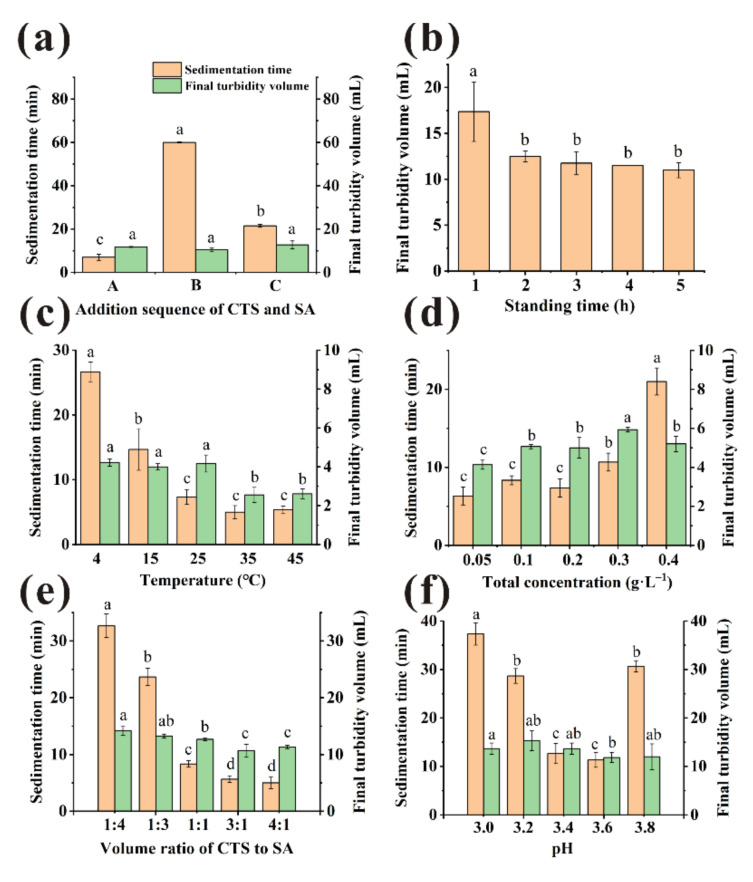
Various factors ((**a**): addition sequence, (**b**): standing time, (**c**): temperature, (**d**): total concentration, (**e**): volume ratios, (**f**): pH) to influence sedimentation time and final turbidity volume. (Different little letters mean a significant difference at the level of 0.05).

As shown in Figure 3a, when sequence A was used, the sedimentation time of clarification achieved a minimum (*p* < 0.05). The sedimentation time was shortened more than 60% for sequence A compared with the single clarified application. Larger flocs were observed to form when treated by sequence A compared with other method, which may facilitate the flocs settling [23]. Considering the above results, the most suitable added sequence of clarifiers was CTS first and then SA. For the purpose of simplicity, we term the sequence A as ‘CTS/SA’. The clarified effect of CTS/SA was much better than using SA or CTS alone, since CTS/SA formed an insoluble network containing the groups of -NH^3+^ and -COO^−^ to remove impurities while CTS or SA only had positive or negative functional groups [10,12].

#### 3.2.2. The Standing Time

The bayberry juice was clarified by CTS/SA with total concentration of 0.2 g/L (CTS:SA = 1:1, *v/v*), then incubated at 25 °C. The results of various standing time were shown in Figure 2b. There were large floccules dispersed in bayberry juice at 0 h, so the transmittance relatively low (0.83 ± 0.66%). The transmittance increased quickly during the first hour, indicating that most suspended substances were sedimented [10]. From 1 h to 5 h, the transmittance of different standing time was not significantly improved (*p* > 0.05). However, the final flocs volume decreased significantly in the first two hours (Figure 3b). Studies indicated that the long-time storage caused decreases of pleasant flavor compounds and changes in pH, solid content, etc. of bayberry juice [25,26]. Considering the clarified effect, efficiency and quality of juice, 2 h was selected as the most appropriate standing time.

#### 3.2.3. The Temperature

CTS/SA was added to the juice with a volume ratio of 1:1, a total concentration of 0.2 g/L and then incubated at varied temperature for 2 h. Temperature had significant effect on the transmittance (Figure 2c). With the increase in temperature, the transmittance was first enhanced and then declined. The reason may be that the increase of temperature accelerated particles collision frequency so larger particles were more easily formed [27]. However, as the temperature further increased, non-enzymatic browning reactions occurred between reducing sugars and amino acid in juice, thereby decreasing the transmittance [28]. Therefore, the maximum of transmittance was obtained at 15 °C. However, there was little difference of transmittance between 15 °C and 25 °C (*p* > 0.05), 91.98 and 91.07%, respectively. Furthermore, the sedimentation time of 25 °C was significantly shorter than 15 °C (Figure 3c), and 25 °C is closest to room temperature. Considering the clarification effect, efficiency, and cost, 25 °C was selected as the most appropriate temperature.

#### 3.2.4. The Total Concentration

To improve transmittance and decrease clarifying agent dosage, effect of the total concentration on transmittance was discussed. Dosage was as a key factor because insufficient clarifiers could not precipitate the impurities completely, while the excessive would form a space protective layer and prevent sedimentation [27]. CTS/SA at a volume ratio of 1:1 of various concentration was tried and incubated at 25 °C for 2 h. As presented in Figure 2d, no significant differences were observed in the range of 0.1–0.3 g/L (*p* > 0.05). Electrostatic forces were provided insufficiently to absorb impurities when dosage of CTS/SA was 0.05 g/L, causing the relatively poor clarification effect [29]. A synergistic effect of CTS/SA can be observed as the effective range of dosage was wider than individual clarifiers. There was no significantly difference between 0.1 g/L and 0.2 g/L on sedimentation time (Figure 3d). Overall, the appropriate total concentration of clarifying agent was 0.1 g/L. 

#### 3.2.5. The Volume Ratio of CTS and SA

CTS/SA at 0.1 g/L of various volume ratio was used and incubated at 25 °C for 2 h. The results were shown in Figure 2e. As the ratio of CTS to SA increased, the transmittance increased but then decreased overall. The protonated amines on CTS and the carboxylate moieties on SA combine through ionic interaction, i.e., CTS bridges with SA [30]. In this process, the impurities such as protein were removed from juice by mechanism of bridging flocculation and charge neutralization. Insufficient dosage of CTS might not conductive to full bridging, and relatively excessive SA would form a colloid network to prevent sedimentation of impurities [10]. Therefore, the sedimentation time when clarified by relatively excess SA was much longer than that ratio of 1:1 (Figure 3e). While too much CTS would quickly form large micelles and precipitate, leading to poor clarification effect. The optimal volume ratio of CTS and SA was 1:1 when the opposite charges could be neutralized best with a maximum transmittance of 90.77 ± 0.66%.

#### 3.2.6. The pH

Electric charge and microscopic morphology of CTS and SA in aqueous solution was influenced by pH, thus clarification effect was affected [27]. Amino groups of chitosan were sufficiently protonated to NH^3+^ and behaved as a typical cationic polyelectrolyte in acidic environment [31]. As shown in Figure 2f, pH range of 3.2–3.8 could be considered suitable for clarification. The sedimentation time of clarification first decreased and then increased with pH rising (Figure 3f). There were no statistical differences (*p* > 0.05) of sedimentation time between samples treated at pH 3.6 and 3.4. Considering pH of unclarified bayberry juice was about 3.4, pH of juice was not adjusted for quality and cost reasons.

#### 3.2.7. Optimization of Clarification Condition by Orthogonal Experiment

On basis of the results of the single factors experiments above, the orthogonal test was conducted using three most influential levels of three factors (Table 1). The influence of different factors on transmittance decreased in the order of volume ratio of CTS to SA > incubation temperature > total concentration according to the R values. The results of variance analysis in Table S2 showed that volume ratio of CTS to SA affected the transmittance (*p* < 0.1). The maximum transmittance was obtained under the combination of A2B2C2, where the volume ratio, concentration and temperature were CTS:SA = 1:1, 0.1 g/L and 25 °C, respectively. Through experimental verification, the transmittance was 91.52 ± 0.65% under the optimal clarified condition. 

Furthermore, the transmittance of juice clarified by NS/CTS/SA was 82.79 ± 0.57% which was lower than clarified by CTS/SA directly. It may be that the reduction of suspended solid after NS made it harder for other small impurities to flocculate through mechanism of bridging and sweeping. The decrease of suspended solid can explain why sedimentation time (5.6 min) was shorter than clarified by CTS/SA directly.

### 3.3. Physicochemical Parameters

#### 3.3.1. Total Carbohydrate and Soluble Solids Evaluation

As seen in Table 2, the contents of carbohydrate and soluble solids in the unclarified juice were 93.48 mg/mL and 7.93 °Brix. After clarification, both values decreased compared with unclarified samples, whereas the total carbohydrate contents and soluble solids were reduced by about 0.25–9.37 mg/mL and 0.13–0.33 °Brix, respectively. Sugars, polyphenols, and proteins could combine to generate unstable complexes, leading to precipitation in the juice [28]. However, there was no significant correlation between transmittance and the contents of soluble solids (r = −0.779, *p* > 0.05), total carbohydrate (r = −0.259, *p* > 0.05), which indicated that increased transmittance is less depended on the removal of them.

#### 3.3.2. Protein and Pectin Contents Analysis

Contents of protein and pectin of samples were reported in Table 2. The protein content of unclarified juice was 478.03 mg/L and was around 30–98% lower after clarification. A previous study reported that some polysaccharides possess affinity to proteins, and chitosan can coagulate the anionic components such as pectin and protein, thus reducing the turbidity of the beverage [32]. The reduction in protein was highest for bayberry juice clarified with CTS/SA which was also highest on transmittance value. Transmittance showed significant correlation (r = −0.877, *p* < 0.05) with protein, which demonstrated that one mechanism for clarification of CTS and SA was probably the removal of protein.

Conventionally, immediate turbidity is presumed to be caused by pectin. The soluble pectin readily leads to clouding of the juice and subsequent precipitation [4]. As shown in Table 2, juice treated by CTS/SA obtained the lowest pectin content of 27.96 mg/L, while the unclarified sample was 198.48 mg/L. Transmittance was strongly negatively correlated with pectin contents (r = −0.895, *p* < 0.05). The significant reduction of pectin contents may be another reason why CTS/SA obtained the best results. NS/CTS/SA had a lower clearance rate of protein and pectin than CTS/SA, owing to the fact that bridging effect was probably weakened after NS treatment, as particles decrease [27].

#### 3.3.3. Total Phenolic Content, Total Anthocyanins Content, Total Flavonoids Content, and Tannin Content

The existence of polyphenols is one of the main reasons for the turbidity of fruit juice [5]. Moreover, the interaction between polyphenols and proteins or the oxidation/polymerization of polyphenols would lead to the formation of haze during storage [33]. Polyphenols include substances such as anthocyanin, flavonoid, tannin, etc. As Table 3 presented, the downward trend of polyphenols content was very obvious compared with unclarified sample. The results were similar to the values measured by Belgheisi et al. [34]. There was no significant (*p* > 0.05) difference on total phenolic content among the samples treated by clarifiers. New study has shown that polysaccharides can inhibit the aggregation of protein-polyphenols [35]. This may be the reason for improving transmittance when treated by polysaccharide-based clarifiers such as CTS and SA.

Unclarified juice contained total anthocyanin content of 85.36 mg/L and 5.8−18.8% of the anthocyanin was lost after clarification. The reduction of anthocyanin in juice was 9.16% for composite clarifiers treatment, being less than that for the single SA treatment and previous related researches. The bayberry juice clarified by individual polysaccharide-based clarifiers was studied by Chen et al. [6], and 18.4−41.2% of the anthocyanin was lost compared with raw bayberry juice. After gelatin/bentonite fining, 10.01% of anthocyanin was lost in bayberry juice [36]. In present research, the decreases in anthocyanin of juice showed notable correlations with reduction of flavonoids (r = 0.872, *p* < 0.05) and phenolic (r = 0.863, *p* < 0.05). Since the total anthocyanin content subjected to NS was statistically comparable to the juice treated with CTS/SA, it indicated that CTS/SA was more conductive to the retention of antioxidant, compared with CTS, SA, and NS/CTS/SA.

The content of flavonoid decreased significantly after clarification which was around 20−83% lower. Through clarifying by CTS/SA, a large number of flavonoids were sedimented compared with NS, from 445.21 to 124.36 mg/L. 

Tannin is a key substance causing turbidity in juice [37]. As seen in the results of NS/CTS/SA or CTS/SA, the decreases in the tannin content were obvious with clarified method of CTS/SA. The correlations between transmittance and the contents of total phenolic (r = −0.831, *p* < 0.05), anthocyanin (r = −0.601, *p* > 0.05), total flavonoids (r = −0.869, *p* < 0.05), and tannin (r = −0.922, *p* < 0.01) were calculated. The results clearly demonstrated that tannins had the most significant effect on the transmittance of bayberry juice. Another mechanism of clarification of CTS and SA was probably the removal of polyphenols, especially tannin compounds. Tannin is negatively charged and can be flocculated by chitosan through charge neutralization mechanism [32]. The mechanism for removing tannin of SA may be bridging and sweeping [38]. The removal rate was improved by CTS/SA which could attribute to the formation of polyelectrolyte complexes that gave full play to the strengths above and CTS/SA precipitated each other thus contributing to efficient flocculation [15].

#### 3.3.4. Color Analysis

Color is one of important parameters which influences the first contact of the human with the product [39]. Therefore, color characteristics (L*, a*, b*) were recorded to determine the difference in color. For unclarified juice, the values of L*, a*, and b* were 30.65, 40.44, and 15.37, respectively (Table 4). L* values, which means lightness, was lower than clarified juice significantly (*p* < 0.05) and there was strong correlation between transmittance and L* (r = 0.948, *p* < 0.01). L* values determined in feijoa juice by Schmidt et al. [40] was increased after clarification, which was consistent with present results. The phenomenon can be explained that the impurity particles in juice were reduced after clarification, thus L* increased. The parameter a* of clarified juice which means color coordinates from green (negative value) to red (positive value) was higher than unclarified sample. Consumers eyes can distinguish the colors between two samples when Δ*E* > 3. Δ*E* of clarified juice ranged from 4.96 (NS) to 20.27 (NS/CTS/SA) compared with raw juice. In a word, the color of sample after clarification was more attractive because the values of L* and a* were increased.

### 3.4. GC-MS Analysis

#### 3.4.1. Comparison of Volatile Flavor Compounds of Bayberry Juice Clarified with Different Methods

In addition to physicochemical properties, the aroma was essential to investigate because of its great impact on consumer acceptance. A total of 71 volatile flavor compounds were found in bayberry juice, including 12 aldehydes, 21 alcohols, 15 esters, 11 terpenes, and 12 others compounds (Table S3). Unclarified, NS, CTS, SA, CTS/SA, NS/CTS/SA contained 53, 48, 51, 51, 61, and 57 volatile compounds, respectively. The dominant volatiles in unclarified juice were caryophyllene (7193.87 μg/L), followed by (E)-2-Nonenal (6097.88 μg/L) and (Z)-3-Nonen-1-ol (2718.57 μg/L). The concentrations of terpenes, especially caryophyllene, were declined after clarification. In contrast, the level of aldehydes, alcohols and esters showed an obvious increase.

#### 3.4.2. Determination of Key Odor Compounds Using ROAV Analysis 

Although a large number of volatile components were detected by GC-MS, only a few of them played a key role in the overall aroma. ROAV method was introduced to further evaluate and confirm the relevant volatile components. Referring to the literature, the aroma thresholds of 43 volatile aroma compounds were found and the results of compounds with ROAV > 0.1 were shown in Table 5. 

(E)-2-nonenal content was relatively high and its aroma threshold value was low, so made a great contribution to the overall aroma. Therefore, ROAV_max_ (100) was defined by the (E)-2-nonenal which can emit green and cucumber odor in all samples except CTS/SA. ROAV_max_ was defined by beta-damascenone in juice clarified by CTS/SA which made it have a unique sweet and green odor. (E)-2-nonenal, 2,4-nonadienal and beta-damascenone were common key odor components (ROAV > 1) in six samples. (E, E)-2,4-nonadienal was the key odor component in all clarified samples with fatty and pungent odor [41]. In addition, 2-heptanol as a key odor component only existed in the juice clarified by NS/CTS/SA which can emit “mushroom” and earthy odor. Nonanal, (E)-2-octenal and 1-penten-3-one could play an important role in modifying the overall odor (0.1 < ROAV < 1) in all samples. Caryophyllene can emit woody odor which played an important role only in unclarified juice [42]. The obvious reduction of caryophyllene content in clarified juice caused the ROAV value below 0.1. Additionally, 3,7-Dimethyl-1,6-octadien-3-ol, which contributed to the flowery odor, could play an embellished role in juice clarified by SA and CTS/SA. (E, Z)-3,6-Nonadien-1-ol could play an important role in juice clarified by SA, CTS/SA, and NS/CTS/SA with ‘‘green and fresh” odor. Hexanoic acid, ethyl ester in juice clarified by CTS/SA with ROAV of 0.125 contributed to fruity and cucumber odor. Further, 2(3H)-Furanone, which could emit sweet and spice odor played an import part in the juice clarified by NS, SA, CTS/SA, and NS/CTS/SA. Additionally, 2-Pentlyfuran with fruity and green odor could play an embellish role in aroma of samples clarified with CTS, SA, and CTS/SA [42].

#### 3.4.3. Principal Component Analysis (PCA) of Key Odor Compounds 

**Figure 4:**
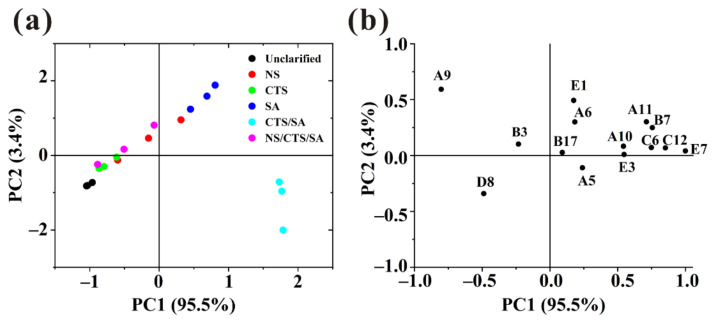
Principal component analysis (PCA) plots of the key odor compounds identified by different clarification methods. (**a**) PC1 vs. PC2 score scatter plot; (**b**) loading weight plot (codes correspond to the compounds described in Table S3).

With the aim of studying the similarities and differences between various samples, the concentration of 14 volatile compounds with ROAV > 0.1 was analyzed using PCA. As shown in Figure 4a, 98.9% of the variability in the bayberry juice could be explained by two principal PCs. PC1 explained 95.5% of the variability. which was the main contributor to the separation of samples. At the same time, the loading plot (Figure 4b) enabled us to visualize the relative importance of each volatile compounds and the possible relationships between different samples and volatile compounds [20]. The load factors of PC1 mainly included beta-damascenone, dihydro-5-pentyl-2(3H)-furanone, 3,7-dimethyl-1,6-octadien-3-ol, hexanoic acid, ethyl ester, indicating that these components were significantly impacted on the discrimination. They gave the contribution to the odor of sweet, green, flower and fruit. Along the direction of PC1, unclarified sample and juice clarified by CTS/SA were far apart, which meant there was a great difference between them and the mentioned compounds content of the latter was higher. It indicated that juice clarified by CTS/SA had a certain improving effect on its aroma. The compounds reflected by PC2 mainly include (E)-2-nonenal and 1-penten-3-one. (E)-2-nonenal gave the contribution to the odor of green and cucumber and 1-penten-3-one can emit the pungent and train oil-like odor which would bring unpleasant feelings to consumers. Along the direction of PC2, unclarified sample and juice clarified by SA were far apart, and there was little difference between unclarified sample and juice treated by CTS/SA. Generally, composite clarifiers can improve the aroma by enhancing the sweet, green, flower, and fruit odor. 

## 4. Conclusions

CTS/SA composite clarifiers as a novel clarified technique used in bayberry juice was investigated. The optimal clarification conditions were obtained when 0.05 g/L CTS was added first then 0.05 g/L SA in juice, standing for 2 h at 25 °C. Under optimal conditions, the transmittance of the juice increased to 91.2% after clarifying with CTS/SA, but only 44.29% and 38.46% were obtained for CTS or SA clarification, respectively. Furthermore, the sedimentation time of CTS/SA-treated juice was about 60% shorter than that of CTS- treated or SA-treated sample. Meanwhile, the juice clarified by CTS/SA obtained a stronger, pleasant odor and more attractive color when compared with the raw bayberry juice and juice treated by single clarifiers. The content of pectin, protein, and polyphenols significantly decreased which may explain why the transmittance of juice increased. Moreover, the strong correlation between tannin and transmittance indicated that the removal of tannin may be the key factor to improve the clarification effect of bayberry juice. Our results suggest that CTS/SA may provide practical knowledge in the production of high-quality bayberry juice.

## Figures and Tables

**Table 1:** Transmittance of bayberry juice treated by composite clarifiers in orthogonal test.

Experimental Number	Volume Ratios of CTS to SA(A)	Total Concentration(B)/g·L^−1^	Incubation Temperature(C)/°C	Transmittance/%
1	3 (3:1)	3 (0.2)	1 (15)	25.17 ± 1.80
2	1 (1:3)	2 (0.1)	3 (35)	38.37 ± 3.72
3	3	1 (0.05)	3	12.06 ± 1.25
4	1	3	2 (25)	49.79 ± 0.87
5	2 (1:1)	3	3	85.47 ± 0.68
6	3	2	2	41.76 ± 1.76
7	2	2	1	83.70 ± 1.02
8	2	1	2	76.23 ± 0.15
9	1	1	1	58.54 ± 2.25
K1	146.71	146.84	167.42	
K2	245.41	163.84	167.79	
K3	78.99	160.43	135.90	
k1	48.90	48.95	55.81	
k2	81.80	54.61	55.93	
k3	26.33	53.48	45.30	
R	55.47	5.67	10.63	

**Table 2:** Effects of different clarification methods on soluble solids, total carbohydrate, protein, and pectin content of bayberry juice.

Samples	Soluble Solids (°Brix)	Total Carbohydrate (mg/mL)	Protein Content (mg/L)	Pectin Content (mg/L)
Unclarified	7.93 ± 0.06 ^a^	93.48 ± 2.42 ^a^	478.03 ± 16.08 ^a^	198.48 ± 3.11 ^a^
NS	7.77 ± 0.06 ^b^	84.11 ± 1.05 ^b^	325.04 ± 3.91 ^b^	179.78 ± 14.00 ^b^
CTS	7.80 ± 0.00 ^b^	84.81 ± 3.17 ^b^	138.25 ± 1.09 ^c^	46.67 ± 7.62 ^d^
SA	7.60 ± 0.00 ^c^	93.23 ± 2.84 ^a^	71.12 ± 1.20 ^d^	70.21 ± 6.02 ^d^
CTS/SA	7.60 ± 0.00 ^c^	84.85 ± 0.30 ^b^	9.06 ± 11.08 ^e^	27.96 ± 1.56 ^e^
NS/CTS/SA	7.60 ± 0.00 ^c^	88.76 ± 1.20 ^ab^	76.4 ± 10.02 ^d^	56.57 ± 4.67 ^d^

Values represent means of triplicate determination ± SD, different letters in the same column represents the significant differences between the values (*p* < 0.05).

**Table 3:** Effects of different clarification methods on total phenolic, total anthocyanins, total flavonoids, and tannin content of bayberry juice.

Samples	Total Phenolic Content (mg/L)	Total Anthocyanins Content (mg/L)	Total Flavonoids Content (mg/L)	Tannin Content (mg/L)
Unclarified	868.94 ± 37.62 ^a^	85.36 ± 2.99 ^a^	556.40 ± 11.90 ^a^	194.78 ± 3.83 ^a^
NS	726.48 ± 6.81 ^b^	80.37 ± 3.04 ^b^	445.21 ± 12.98 ^b^	158.41 ± 17.34 ^b^
CTS	481.00 ± 5.36 ^c^	75.49 ± 0.21 ^c^	156.75 ± 6.94 ^c^	109.33 ± 2.09 ^c^
SA	470.89 ± 3.65 ^c^	69.30 ± 1.77 ^d^	133.79 ± 4.35 ^d^	113.52 ± 25.42 ^c^
CTS/SA	467.09 ± 1.95 ^c^	77.54 ± 0.99 ^bc^	124.36 ± 7.51 ^d^	87.99 ± 16.90 ^c^
NS/CTS/SA	465.93 ± 2.59 ^c^	70.10 ± 0.80 ^d^	92.35 ± 12.56 ^e^	84.71 ± 10.87 ^c^

Values represent means of triplicate determination ± SD, different letters in the same column represents the significant differences between the values (*p* < 0.05).

**Table 4:** Effects of different clarification methods on color parameters of bayberry juice.

Samples	L*	a*	b*	Δ*E*
Unclarified	30.65 ± 0.03 ^e^	40.44 ± 0.026 ^d^	15.37 ± 0.04 ^b^	–
NS	35.06 ± 0.09 ^d^	41.46 ± 0.04 ^c^	17.40 ± 0.06 ^a^	4.96 ± 0.05 ^d^
CTS	42.21 ± 0.11 ^c^	43.45 ± 0.04 ^a^	12.77 ± 0.18 ^d^	12.23 ± 0.14 ^c^
SA	45.80 ± 0.69 ^b^	40.86 ± 0.75 ^d^	14.01 ± 0.80 ^c^	15.24 ± 0.73 ^b^
CTS/SA	50.26 ± 0.11 ^a^	42.56 ± 0.13 ^b^	12.42 ± 0.13 ^de^	19.94 ± 0.11 ^a^
NS/CTS/SA	50.61 ± 0.24 ^a^	41.60 ± 0.20 ^c^	12.03 ± 0.22 ^e^	20.27 ± 0.26 ^a^

Values represent means of triplicate determination ± SD, different letters in the same column represents the significant differences between the values (*p* < 0.05).

**Table 5:** Volatile compounds with ROAV ≥ 0.1 in different clarification methods of bayberry juice.

				ROAV					
Serial Number	Compound	Odor Descriptor ^a^	Aroma Threshold ^b^ (μg/kg)	Unclarified	NS	CTS	SA	CTS/SA	NS/ CTS/SA
A5	Nonanal	sweet, green, fruity	1	0.296	0.218	0.200	0.293	0.384	0.393
A6	(E)-2-Octenal	fatty	3	0.329	0.508	0.688	0.552	0.482	0.365
A9	(E)-2-Nonenal	green, cucumber	0.08	100.000	100.000	100.000	100.000	81.678	100.000
A10	2,4-Nonadienal	deep-fried	0.05	1.901	1.903	1.927	2.881	2.989	2.168
A11	(E,E)-2,4-Nonadienal	fatty, pungent	0.09	0.000	4.251	5.110	8.702	9.582	7.055
B3	2-Heptanol	mushroom, earthy	0.07	0.000	0.000	0.000	0.000	0.000	1.241
B7	3,7-Dimethyl-1,6-Octadien-3-ol	flowery	6	0.044	0.061	0.078	0.112	0.117	0.088
B17	(E,Z)-3,6-Nonadien-1-ol	green, fresh	3	0.086	0.051	0.046	0.119	0.121	0.139
C6	Hexanoic acid, ethyl ester	fruity, cucumber	1	0.044	0.073	0.000	0.099	0.125	0.052
C12	Dihydro-5-pentyl-2(3H)-furanone	sweet, spice	25	0.045	0.116	0.098	0.180	0.251	0.170
D8	Caryophyllene	woody	64	0.147	0.026	0.004	0.007	0.004	0.006
E1	1-Penten-3-one	pungent, train oil-like	1	0.132	0.123	0.166	0.196	0.136	0.132
E3	2-Pentylfuran	fruity, green	6	0.025	0.044	0.130	0.110	0.135	0.086
E7	beta-Damascenone	sweet, green	0.002	24.85	32.31	43.351	77.321	100.000	49.250

^a^ Odor descriptor was obtained according to literature: (1) Cheng et al. [42] (2) Zhang et al. [43] (3) Olivares et al. [44] (4) Yu et al. [41] (5) Morales et al. [45] (6) Sellami et al. [46]. ^b^ Aroma threshold was determined in water by according to literature: Sun et al. [47].

## Data Availability

The data presented in this study are available on request from the corresponding author. The data are not publicly available due to privacy reasons.

## Supplementary Materials

The following supporting information can be downloaded at: https://www.mdpi.com/article/10.3390/foods11050671/s1, Figure S1: Flow chart of Chinese bayberry juice processing and clarification.; Table S1: Factors and specific ranges of parameters in orthogonal experiments for clarification technology optimization.; Table S2: Variance analysis of orthogonal experiment.; Table S3: The composition and concentrations of the volatile compounds detected in bayberry juice under different clarification method.
