# Supplementary material for: Optimized Clarification Technology of Bayberry Juice by Chitosan/Sodium Alginate and Changes in Quality Characteristics during Clarification
Source: Foods. 2022 Feb 24;11(5):671. doi: 10.3390/foods11050671 (PMC8909023; doi:10.3390/foods11050671)
Supplement: Supplementary file 1 [file foods-11-00671-s001.zip › foods-1584065-supplementary.pdf]

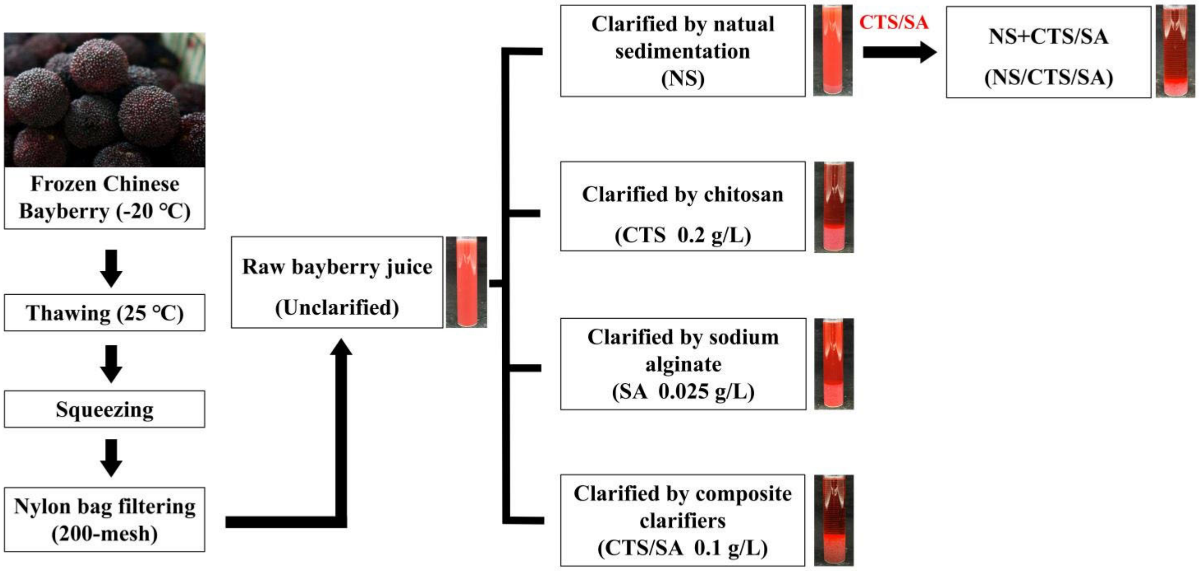

Figure S1. Flow chart of Chinese bayberry juice processing and clarification.

Table S1. Factors and specific ranges of parameters in orthogonal experiments for clarification technology optimization.

Level	Factors		
	A	B	C
	Volume ratios of CTS to SA	Total concentration ($g \cdot L^{-1}$)	Incubation temperature (°C)
1	1:3	0.05	15
2	1:1	0.1	25
3	3:1	0.2	35

Note: Values represent means of triplicate determination ± SD, different letters in the same column represents the significant differences between the values ($p < 0.05$).

Table S2. Variance analysis of orthogonal experiment.

Factor	Sum of squares	Freedom	Mean square error	F	*P*
A	4668.89	2	2334.45	11.15	a
B	53.93	2	26.97	0.13	b
C	223.37	2	111.68	0.53	b
Error	418.62	2	209.31		

Note F(0.1)=9.00, F(0.05)=19

a. $p < 0.1$

b. $p > 0$.

Table S3. The composition and concentrations of the volatile compounds detected in bayberry juice under different clarification method.

Code	RI	CAS	Compound	Formula	Concentration/μg/L (mean±SD)					
					Unclarified	NS	CTS	SA	CTS/SA	NS/CTS/SA
Aldehydes										
A1	1115	000066-25-1	Hexanal	$C_6H_{12}O$	146.51±56.84	201.82±38.76	207.98±19.05	140.58±7.42	125.78±22.45	116.56±8.74
A2	1152	001576-87-0	Trans-2-Pentenal	$C_6H_{12}O$	122.46±26.77	134.58±30.81	114.09±8.85	108.48±8.10	70.78±12.67	79.92±12.43
A3	1200	000111-71-7	Heptanal	$C_7H_{14}O$	9.30±3.47	nd	5.94±1.30	nd	16.42±8.35	nd
A4	1327	018829-55-5	(E)-2-Heptenal	$C_7H_{12}O$	82.04±2.83	72.16±15.23	80.08±9.47	nd	42.42±5.47	40.58±3.15
A5	1395	000124-19-6	Nonanal	$C_9H_{18}O$	225.30±66.72	197.58±67.79	159.67±14.95	168.75±35.60	190.09±89.12	230.59±69.51
A6	1430	002548-87-0	(E)-2-Octenal	$C_8H_{14}O$	751.25±233.17	1379.63±438.88	1649.37±176.43	956.07±50.35	715.13±121.28	642.53±51.95
A7	1451	002277-16-9	(E)-4-Nonenal	$C_9H_{16}O$	320.37±45.65	3796.56±716.31	2497.06±236.29	1579.13±157.60	302.23±65.83	1823.11±384.31
A8	1518	000100-52-7	Benzaldehyde	C_7H_6O	nd	446.25±69.05	275.80±30.03	383.94±74.52	244.98±122.57	268.05±31.04
A9	1537	018829-56-6	(E)-2-Nonenal	$C_9H_{16}O$	6097.88±408.78	7238.27±352.31	6391.89±713.08	4614.87±297.14	3231.61±715.47	4699.73±226.19
A10	1659	006750-03-4	2,4-Nonadienal	$C_9H_{14}O$	72.45±16.40	86.10±21.55	77.00±3.46	83.09±30.85	73.92±32.17	63.67±5.60
A11	1698	005910-87-2	(E,E)-2,4-Nonadienal	$C_9H_{14}O$	nd	346.14±58.97	367.42±32.40	451.79±122.18	426.50±92.66	373.03±67.32
A12	1804	015764-16-6	2,4-Dimethylbenzaldehyde	$C_9H_{10}O$	nd	nd	nd	46.05±25.40	nd	53.54±23.74
Total					7827.56±860.64^{b}	13899.09±1809.66^{a}	11826.29±1245.32^{a}	8532.75±809.16^{b}	5439.87±1288.05^{c}	8391.31±883.99^{b}

Alcohols									
B1	1226	000123-51-3	3-methyl-1-Butanol	$C_5H_{12}O$	22.27±3.63	91.47±16.11	36.50±7.36	109.34±8.86	99.78±6.05	107.42±9.33
B2	1265	000071-41-0	1-Pentanol	$C_5H_{12}O$	nd	nd	50.63±8.08	nd	nd	nd
B3	1330	000543-49-7	2-Heptanol	$C_7H_{16}O$	nd	nd	nd	nd	nd	51.03±4.69
B4	1362	000111-27-3	1-Hexanol	$C_6H_{14}O$	144.35±36.19	232.20±41.95	133.63±20.18	205.27±16.40	211.29±12.15	223.03±16.51
B5	1390	000928-96-1	(Z)-3-Hexen-1-ol	$C_6H_{12}O$	116.13±21.01	193.16±29.37	98.66±16.30	134.29±9.55	161.53±7.02	125.00±12.66
B6	1428	000123-96-6	2-Octanol	$C_8H_{18}O$	145.36±15.21	399.37±143.61	nd	521.85±12.21	421.84±23.00	439.55±54.08
B7	1551	000078-70-6	3,7-dimethyl-1,6-Octadien-3-ol	$C_{10}H_{18}O$	200.29±50.44	330.85±100.65	374.93±36.15	387.00±43.94	346.09±16.39	310.30±38.28
B8	1563	000111-87-5	1-Octanol	$C_8H_{18}O$	263.33±60.16	434.34±122.05	238.37±28.16	558.44±103.41	451.29±40.71	524.62±68.33
B9	1565	029803-81-4	2-Cyclohexen-1-ol, 1-methyl-4-(1-methylethyl)-, trans-	$C_{10}H_{18}O$	nd	nd	nd	nd	72.22±8.91	nd
B10	1604	000562-74-3	Terpinen-4-ol	$C_{10}H_{18}O$	2271.03±82.35	1884.00±167.61	1125.73±158.41	5506.10±278.34	2323.29±209.54	4766.24±454.46
B11	1630	029803-82-5	2-Cyclohexen-1-ol, 1-methyl-4-(1-methylethyl)-, cis-	$C_{10}H_{18}O$	nd	nd	nd	58.55±3.33	38.04±4.62	nd
B12	1642	002216-51-5	Levomenthol	$C_{10}H_{20}O$	118.30±47.11	nd	nd	nd	45.66±11.57	62.78±3.50
B13	1691	010340-23-5	(Z)-3-Nonen-1-ol	$C_9H_{18}O$	2718.57±210.69	5372.78±367.61	3801.59±335.96	7173.91±427.04	9117.26±1045.76	6406.39±599.54
B14	1703	000507-70-0	Borneol	$C_{10}H_{18}O$	219.31±26.71	71.68±19.67	118.56±5.73	nd	135.15±42.88	nd
B15	1717	031502-14-4	(E)-2-Nonen-1-ol	$C_9H_{18}O$	345.77±136.00	134.19±63.77	208.80±24.56	255.59±60.60	99.90±12.71	368.76±63.96
B16	1720	035854-86-5	(Z)-6-Nonen-1-ol	$C_9H_{18}O$	487.56±304.00	203.95±66.28	181.42±26.85	340.61±55.69	251.83±36.85	380.41±50.16
B17	1735	056805-23-3	(E,Z)-3,6-Nonadien-1-ol	$C_9H_{16}O$	195.55±128.55	139.34±37.25	109.09±12.59	205.11±27.13	179.60±21.67	244.57±32.16
B18	1748	016721-38-3	cis-Piperitol	$C_{10}H_{18}O$	nd	nd	nd	nd	26.06±5.49	nd
B19	1909	000060-12-8	Phenylethyl alcohol	$C_8H_{10}O$	134.09±30.87	595.48±145.96	149.63±21.10	547.57±65.48	624.33±63.66	565.57±25.95
B20	>2000	000122-97-4	3-Phenylpropanol	$C_9H_{12}O$	46.23±8.94	92.80±5.63	36.35±1.33	80.93±20.99	110.08±14.40	85.00±8.27
B21	>2000	000077-53-2	Cedrol	$C_{15}H_{26}O$	nd	nd	nd	22.65±1.76	nd	nd
Total					7434.13±1161.85^{c}	10175.60±1327.88^{b}	6663.89±702.74^{c}	16107.22±1134.74^{a}	14715.24±1583.37^{a}	14659.66±1441.88^{a}
Esters										

C1	<1000	000141-78-6	Ethyl acetate	$C_4H_8O_2$	77.14±17.74	178.07±52.60	182.11±15.75	191.98±32.23	245.58±37.62	172.47±26.89
C2	1006	000554-12-1	Methyl propionate	$C_4H_8O_2$	46.07±16.75	nd	nd	nd	nd	nd
C3	1014	000547-63-7	Propanoic acid, 2-methyl-, methyl ester	$C_5H_{10}O_2$	nd	nd	nd	nd	14.83±3.32	nd
C4	1119	000624-24-8	Methyl valerate	$C_6H_{12}O_2$	11.42±4.86	24.01±5.66	19.31±2.93	nd	17.84±4.38	18.72±2.60
C5	1211	000106-70-7	methyl hexanoate	$C_7H_{14}O_2$	140.17±39.53	325.17±53.73	112.54±13.96	113.77±4.88	15.26±33.07	87.74±10.12
C6	1258	000123-66-0	Hexanoic acid, ethyl ester	$C_8H_{16}O_2$	33.70±6.07	65.60±33.93	nd	56.97±18.71	61.61±20.92	30.43±2.75
C7	1269	013894-62-7	3-Hexenoic acid, methyl ester, (Z)-	$C_7H_{12}O_2$	212.78±47.71	285.14±86.03	131.79±19.89	115.19±8.97	175.03±33.04	90.90±10.22
C8	1501	007367-81-9	Methyl trans-2-octenoate	$C_9H_{16}O_2$	nd	112.97±19.05	51.77±6.24	76.68±11.82	52.61±48.30	66.15±8.92
C9	1618	000093-58-3	Benzoic acid, methyl ester	$C_8H_8O_2$	676.92±62.34	1080.43±118.16	1065.33±170.74	987.63±79.29	829.36±91.12	825.41±43.53
C10	1769	000119-36-8	Methyl salicylate	$C_8H_8O_3$	317.95±21.19	332.10±86.09	342.48±45.16	323.80±20.43	242.47±22.58	300.73±31.26
C11	1842	000103-25-3	Methyl 3-phenylpropanoate	$C_{10}H_{12}O_2$	34.39±17.75	59.58±11.52	28.70±5.83	36.03±8.69	53.24±6.66	33.81±6.67
C12	>2000	000104-61-0	2(3H)-Furanone, dihydro-5-pentyl-	$C_9H_{16}O_2$	856.57±215.88	2630.40±502.23	1954.16±301.15	2602.37±362.47	3103.98±530.24	2490.62±260.85
C13	>2000	000103-36-6	Ethyl cinnamate	$C_{11}H_{12}O_2$	nd	nd	nd	33.88±5.66	30.88±4.05	25.77±9.34
C14	>2000	000628-97-7	Hexadecanoic acid, ethyl ester	$C_{18}H_{36}O_2$	13.09±22.67	nd	nd	nd	nd	nd
C15	>2000	000084-74-2	Dibutyl phthalate	$C_{16}H_{22}O_4$	17.57±7.26	24.17±9.64	38.37±12.06	70.13±20.80	33.48±17.08	35.95±14.78
Total					2437.77±479.75^{b}	5117.64±978.63^{a}	3926.55±593.72^{a}	4608.41±573.95^{a}	5016.17±852.38^{a}	4178.69±427.92^{a}
Terpenes										
D1	1129	040087-62-5	1,3-cis,5-cis-Octatriene	$C_{10}H_{16}$	nd	nd	nd	nd	22.30±3.22	14.77±3.73
D2	1178	000099-83-2	α-Phellandrene	$C_{10}H_{16}$	23.16±5.70	110.07±39.01	50.68±2.05	75.20±5.92	216.94±15.30	62.39±6.06
D3	1191	000099-86-5	α-terpinene	$C_{10}H_{16}$	nd	112.51±69.57	143.41±20.81	155.40±18.97	403.17±36.13	132.61±13.80
D4	1209	005989-27-5	D-Limonene	$C_{10}H_{16}$	30.54±8.85	84.34±33.91	18.58±3.21	118.92±54.43	22.29±6.81	67.23±6.39
D5	1217	000555-10-2	β-Phellandrene	$C_{10}H_{16}$	61.79±16.57	nd	97.52±6.80	107.89±17.20	281.43±30.15	104.55±8.41
D6	1264	000100-42-5	phenylethylene	C_8H_8	75.51±28.56	259.67±74.56	67.18±11.90	186.46±14.21	218.57±50.08	149.30±13.60
D7	1288	000586-62-9	Cyclohexene, 1-methyl-4-(1-methylethylidene)-	$C_{10}H_{16}$	15.77±4.59	nd	nd	27.24±6.13	24.57±7.25	23.58±3.33
D8	1594	000087-44-5	Caryophyllene	$C_{15}H_{24}$	7193.87±434.40	1513.10±568.29	229.40±72.30	261.22±95.50	134.91±84.57	242.89±142.44

D9	1666	006753-98-6	Humulene	$C_{15}H_{24}$	669.65±162.00	nd	nd	nd	nd	nd
D10	1711	000502-61-4	α-Farnesene	$C_{15}H_{24}$	27.71±14.86	nd	nd	nd	nd	nd
D11	1978	001139-30-6	Caryophyllene oxide	$C_{15}H_{24}O$	647.44±181.29	592.43±185.95	231.53±37.11	338.13±83.65	217.62±57.42	226.24±50.38
Total					8745.44±1032.15[a]	2672.11±988.88[b]	838.30±154.18[c]	1270.45±296.01[c]	1541.78±290.93[c]	1023.55±248.15[c]
Others										
E1	1070	001629-58-9	1-Penten-3-one	C_5H_8O	100.45±33.17	110.88±35.57	132.40±6.75	112.99±9.05	67.15±12.13	77.76±9.51
E2	1194	000470-67-7	1,4-Cineole	$C_{10}H_{18}O$	nd	nd	nd	nd	nd	33.81±5.22
E3	1254	003777-69-3	2-pentylfuran	$C_9H_{14}O$	113.25±28.46	236.56±65.60	621.96±97.75	381.84±29.02	399.61±52.30	303.64±29.50
E4	1276	000527-84-4	o-Cymene	$C_{10}H_{14}$	107.75±68.69	68.87±21.32	44.01±22.48	124.14±18.38	107.28±20.43	109.75±9.01
E5	1292	000111-13-7	2-Octanone	$C_8H_{16}O$	101.23±12.41	203.18±70.71	79.29±15.80	nd	223.01±39.43	nd
E6	1479	010458-14-7	5-Methyl-2-(1-methylethyl)cyclohexanone,	$C_{10}H_{18}O$	128.57±5.17	nd	nd	nd	nd	nd
E7	1829	023726-93-4	β-Damascenone	$C_{13}H_{18}O$	37.89±1.43	58.47±11.14	69.27±4.95	89.21±13.60	98.91±12.06	57.87±12.23
E8	>2000	002785-89-9	4-Ethyl-2-methoxyphenol	$C_9H_{12}O_2$	122.73±3.60	305.12±60.13	182.19±16.55	426.56±22.09	20.82±4.93	413.52±72.67
E9	>2000	000108-39-4	3-Methylphenol	C_7H_8O	nd	nd	nd	nd	61.51±26.13	56.68±8.25
E10	>2000	000123-07-9	4-Ethylphenol	$C_8H_{10}O$	67.84±10.82	137.26±28.52	52.13±9.85	197.64±19.90	154.53±16.89	193.09±18.53
E11	>2000	000112-05-0	Nonanoic acid	$C_9H_{18}O_2$	nd	nd	24.21±7.28	nd	24.50±7.22	17.89±5.46
E12	>2000	001200-93-7	1H-2-Benzopyran-1-one, 3,4-dihydro-8-hydroxy-3-methyl-	$C_{10}H_{10}O_3$	3.77±6.54	69.26±21.24	19.64±4.85	45.20±2.88	90.45±11.70	48.27±6.14
Total					783.48±170.28[b]	1189.60±314.22[a]	1225.11±186.26[a]	1377.68±114.93[a]	1247.77±203.22[a]	1312.28±176.50[a]

Note: Values represent means of triplicate determination ± SD, different letters in the same column represents the significant differences between the values ($p < 0.05$)
